# *Drosophila* Carrying *Pex3* or *Pex16* Mutations Are Models of Zellweger Syndrome That Reflect Its Symptoms Associated with the Absence of Peroxisomes

**DOI:** 10.1371/journal.pone.0022984

**Published:** 2011-08-03

**Authors:** Minoru Nakayama, Hiroyasu Sato, Takayuki Okuda, Nao Fujisawa, Nozomu Kono, Hiroyuki Arai, Emiko Suzuki, Masato Umeda, Hiroyuki O. Ishikawa, Kenji Matsuno

**Affiliations:** 1 Genome and Drug Research Center, Tokyo University of Science, Noda, Chiba, Japan; 2 Department of Biological Science and Technology, Tokyo University of Science, Noda, Chiba, Japan; 3 Research Institute for Science and Technology, Tokyo University of Science, Noda, Chiba, Japan; 4 Graduate School of Pharmaceutical Science, University of Tokyo, Hongo, Bunkyo-ku, Tokyo, Japan; 5 Structural Biology Center, National Institute of Genetics, and Department of Genetics, The Graduate University for Advanced Studies, Mishima, Shizuoka, Japan; 6 Institute for Chemical Research, Kyoto University, Uji, Kyoto, Japan; Yale School of Medicine, United States of America

## Abstract

The peroxisome biogenesis disorders (PBDs) are currently difficult-to-treat multiple-organ dysfunction disorders that result from the defective biogenesis of peroxisomes. Genes encoding Peroxins, which are required for peroxisome biogenesis or functions, are known causative genes of PBDs. The human peroxin genes *PEX3* or *PEX16* are required for peroxisomal membrane protein targeting, and their mutations cause Zellweger syndrome, a class of PBDs. Lack of understanding about the pathogenesis of Zellweger syndrome has hindered the development of effective treatments. Here, we developed potential *Drosophila* models for Zellweger syndrome, in which the *Drosophila pex3* or *pex16* gene was disrupted. As found in Zellweger syndrome patients, peroxisomes were not observed in the homozygous *Drosophila pex3* mutant, which was larval lethal. However, the *pex16* homozygote lacking its maternal contribution was viable and still maintained a small number of peroxisome-like granules, even though PEX16 is essential for the biosynthesis of peroxisomes in humans. These results suggest that the requirements for pex3 and pex16 in peroxisome biosynthesis in *Drosophila* are different, and the role of PEX16 orthologs may have diverged between mammals and *Drosophila*. The phenotypes of our Zellweger syndrome model flies, such as larval lethality in *pex3*, and reduced size, shortened longevity, locomotion defects, and abnormal lipid metabolisms in *pex16*, were reminiscent of symptoms of this disorder, although the *Drosophila pex16* mutant does not recapitulate the infant death of Zellweger syndrome. Furthermore, *pex16* mutants showed male-specific sterility that resulted from the arrest of spermatocyte maturation. *pex16* expressed in somatic cyst cells but not germline cells had an essential role in the maturation of male germline cells, suggesting that peroxisome-dependent signals in somatic cyst cells could contribute to the progression of male germ-cell maturation. These potential *Drosophila* models for Zellweger syndrome should contribute to our understanding of its pathology.

## Introduction

The peroxisome biogenesis disorders (PBDs) are human recessive hereditary diseases that arise from mutations in *PEX* genes, which encode the Peroxins, essential proteins for the biogenesis of peroxisomes [1]. Currently, 13 *PEX* genes have been identified as causative genes for PBDs [1], [2]. The hallmark of PBDs is the malformation or complete absence of peroxisomes in patients' cells [2]. PBD patients exhibit multiple organ dysfunctions, including developmental and progressive neurological defects, and those with the most severe manifestation, Zellweger syndrome, usually die before they are 1 year old [3]. However, although the disruption of peroxisome functions is thought to be the direct or indirect cause of these disorders, a better understanding of the pathogenesis of PBDs is needed to develop effective treatments.

Mouse models for PBDs, in which *Pex2* [4], *Pex5* [5], *Pex7* [6], or *Pex13* [7] is disrupted, have been developed. Mice homozygous for any of these mutants show various defects that are similar to the symptoms of Zellweger syndrome [8]. However, although important insights into the pathogenesis of PBDs have been obtained from studies of these mouse models, the cellular and molecular bases of the PBD-associated defects are still elusive.

Peroxisomes participate in diverse metabolic processes, including the β-oxidation of VLCFAs (very long chain fatty acids), oxidation of phytanic acid, biosynthesis of ether-phospholipids, and H_2_O_2_ metabolism [9], [10]. Therefore, PBD patients show increased levels of VLCFAs and reduced levels of a polyunsaturated fatty acid, docosahexaenoic acid (DHA), and of plasmalogens (ether-phospholipids). These altered lipid levels are among the diagnostic markers for PBDs [2]. However, the links between the change in lipid content, abnormal cellular functions, and the development of PBDs are unclear.

Classically, peroxisomes were thought to arise through the growth and division of preexisting peroxisomes [11]. However, accumulating evidence supports an ER-dependent mode of peroxisome biogenesis (*de novo* biogenesis), especially in yeast [12], [13], [14] and plants [15], [16]. In addition, a recent report showed that peroxisomes also arise *de novo* from the ER in mammalian cells [17]. Therefore, the existence of two pathways for peroxisome proliferation, the growth and fission pathway and the *de novo* biogenesis pathway, are now largely accepted [18].

The functions of three genes, *Pex3*, *Pex16*, and *Pex19*, are reported to be essential for the *de novo* biogenesis of peroxisomes [17], [19], in which their products contribute to peroxisomal membrane protein targeting [20], [21]. On the other hand, *Pex1*, *Pex2*, *Pex5*, *Pex6*, *Pex7*, *Pex10*, *Pex12*, *Pex13*, and *Pex14* are required for peroxisomal matrix protein import [22], [23]. Recently, *pex2* and *pex10 Drosophila* mutants were reported [24]. The analysis of these mutants revealed that the import of some peroxisomal matrix proteins is required for spermatogenesis and the metabolism of VLCFAs [24]. However, these mutants do not show other phenotypes reminiscent of PBD symptoms, such as defects in neuronal development and function [24]. These results suggested that some level of peroxisome activity is still maintained in the *pex2* and *pex10* mutants. In contrast, the mutation of genes involved in the peroxisomal membrane protein targeting, including *Pex3*, *Pex16*, and *Pex19*, has not been reported in *Drosophila*. *Pex3* and *Pex16* encode integral membrane proteins: PEX16 is a receptor for PEX3, which acts as a docking receptor for incoming peroxisomal membrane proteins [21]. PEX19 binds nascent peroxisomal membrane proteins (PMPs) in the cytoplasm and targets them to PEX3 on the peroxisomal membrane [25].

Here we describe the *Drosophila* lines bearing mutations in the *pex3* and *pex16* genes. In contrast to the *pex2* and *pex10* mutants, which show developmental defects only in spermatogenesis [24], the *pex3* mutant was larval lethal, and the *pex16* mutant showed a reduced lifespan and various defects in development and neural function. We conclude that these mutants reflect broad symptoms of PBDs, and can be viewed as *Drosophila* models of these diseases, especially of Zellweger syndrome, although the *Drosophila pex16* mutant does not recapitulate the infant death seen in Zellweger syndrome patients.

## Results

### *pex3* and *pex16* are the *Drosophila* lines bearing mutant *pex* genes

**Figure 1:**
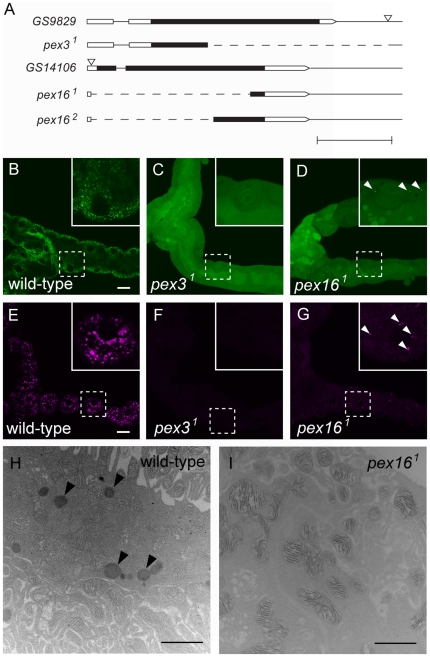
*pex3^1^* and *pex16^1^* lack normal peroxisomes. (A) Genomic lesions induced in *pex3* and *pex16*. Untranslated regions and coding regions are shown as open and filled boxes, respectively. The P-element insertion sites in *GS9829* and *GS14106* are indicated by triangles. Genomic regions deleted in the mutants are indicated by dashed lines. Scale bar represents 0.5 Kb. (B–G) Peroxisomes detected *in vivo*. Malpighian tubules dissected from the larvae of wild-type (B, E), *pex3^1^* homozygotes (C, F), and *pex16^1^* homozygotes (D, G), ubiquitously expressing *UAS-EGFP-SKL* (B–D) or *UAS-PMP70-ECFP* (E–G) driven by *Act-GAL4* are shown. Insets at upper right are higher-magnification images of the area enclosed by broken-line squares. Scale bars in B and E represent 10 µm. (H and I) Electron micrographs of adult malpighian tubule sections stained with DAB in wild-type (H) and *pex16^1^* homozygous flies (I). Arrowheads in H indicate DAB-stained peroxisomes. Scale bar in H represents 1 µm.

To understand the roles of *Drosophila Pex* genes required for peroxisomal membrane protein targeting, we developed loss-of-function mutants of the *pex3* (*CG6859*) and *pex16* (*CG3947*) genes [24]. Using the P-element imprecise excision approach, we isolated deletion mutants of *pex3* and *pex16* (Figure 1A). *pex3^1^* is a deletion mutant lacking the 3′-region of the *pex3* locus, which is therefore missing two-thirds of its coding region (Figure 1A). To confirm that the mRNA corresponding to the deleted genomic region was not synthesized in the *pex3^1^* homozygote, we performed reverse transcription polymerase chain reaction (RT-PCR). No RT-PCR product originating from the deleted genomic regions was detected in the *pex3^1^* homozygote, whereas a product from an intact region was detected (Figure S1). In *pex16^1^* and *pex16^2^*, most of the coding region was deleted (Figure 1A). We also confirmed that no mRNA corresponding to the deleted genomic region in *pex16^1^* was synthesized in the *pex16^1^* homozygote (Figure S1). Based on the molecular lesions of these mutants, it is likely that they were all null alleles. *pex3^1^* homozygotes and trans-heterozygotes of *pex3^1^* and a deletion mutant of the entire *pex3* locus, Df(3L)ED218, died as larvae (data not shown). However, *pex16^1^* homozygotes, trans-heterozygotes of *pex16^1^* and a deletion mutant of the *pex16* locus, Df(3L)ri-XT1, and *pex16^1^* homozygotes lacking the maternal contribution were viable (data not shown).

### *pex3^1^* and *pex16^1^* homozygotes lack normal peroxisomes

The absence of peroxisomes in skin biopsy samples is one of the crucial diagnostic markers of PBDs [1], [2]. Therefore, to evaluate whether our *pex3* and *pex16* mutants could be considered as *Drosophila* models of PBDs, we first confirmed that they lacked peroxisomes. To visualize peroxisomes *in vivo*, we overexpressed proteins with peroxisome-targeting signals. One, EGFP-SKL is an EGFP derivative tagged with the SKL peptide, also designated as PTS1 (peroxisomal targeting signal-1) [26]. The other, PMP70-ECFP, is a chimeric protein of ECFP and PMP70, whose mammalian orthologue localizes to the peroxisomal membrane via its mPTS (peroxisomal membrane-targeting signal) [25]. We co-expressed *EGFP-SKL* with *PMP70-ECFP* in *Drosophila* S2 cells and found that the two proteins were colocalized at small cytoplasmic granules (Figure S2), even though each protein carried a different peroxisome-targeting signal. Therefore, we concluded that these intracellular particles were *Drosophila* peroxisomes.

To determine whether peroxisomes were absent from *pex3^1^* or *pex16^1^* homozygotes, we ubiquitously expressed *UAS-EGFP-SKL* or *UAS-PMP70-ECFP* in the mutant homozygotes or wild-type flies using the GAL4/UAS system (driven by *Act*-GAL4) [27]. In wild-type flies, in the cells of the larval malpighian tubule, EGFP-SKL and PMP70-ECFP specifically localized to peroxisomes (Figure 1B and 1E). However, the EGFP-SKL-positive or the PMP70-ECFP-positive peroxisomes were absent in the same cells in the *pex3^1^* homozygote (Figure 1C and 1F), although a small number of granules (less than one in 10 cells) labeled by PMP70-ECFP, the nature of which was unknown, were detected in these cells (data not shown). These results suggested that *pex3* is essential for the presence of peroxisomes.

In contrast to the *pex3* mutant, the malpighian tubule cells of the *pex16^1^* homozygotes still contained peroxisome-like granules detected by EGFP-SKL and PMP70-ECFP, although their number was greatly reduced compared with wild-type (Figure 1D and 1G). To determine the nature of these peroxisome-like granules in *pex16^1^*, we stained the tissues of wild-type and *pex16^1^* homozygotes with 3, 3′-diaminobenzidine (DAB) and observed them by transmission electron microscopy. In the wild-type malpighian tubules, peroxisomes were detectable by DAB staining (Figure 1H) [28]. In contrast, the *pex16^1^* homozygotes did not contain cells with DAB-positive organelles (Figure 1I). Therefore, although some peroxisomes apparently remained in the *pex16* mutants, they were present at only a very low frequency or they had lost their normal enzymatic activity, which made them undetectable by the DAB reaction.

*pex3* was a recessive lethal mutant as mentioned above, whereas *pex16^1^* homozygotes lacking the maternal contribution could develop to adulthood, and a small number of peroxisome-like granules were still found in them (data not shown). These results suggested that a small number of peroxisomes are still maintained in the absence of PEX16 in *Drosophila*. The *pex16* mutant of the yeast *Yarrowia lipolytica* contains peroxisome-like structures [29]. Therefore, similar to our finding using *Drosophila pex16^1^*, *pex16* is not essential for the presence of peroxisomes in *Yarrowia lipolytica* [29]. In contrast, both PEX3 and PEX16 are known to be essential for the presence of peroxisomes in mammals [30], [31]. Thus, the requirement of Pex16 orthologs in peroxisome biosynthesis may be different among species.

In *Yarrowia lipolytica*, an overexpression of its *pex16* ortholog results in fewer but enlarged peroxisomes [29]. Therefore, if the function of *pex16* orthologs is conserved between yeast and *Drosophila*, we thought that the overexpression of *Drosophila pex16* would give similar defects in peroxisomes in *Drosophila*. To address this, we overexpressed *UAS-pex16* and *UAS-EGFP-SKL* in wild-type flies, and found that the peroxisomes labeled by EGFP-SKL became fewer and larger in the malpighian tubule cells or spermatocytes overexpressing *pex16*, compared with wild-type (Figure S3 A–D′). Quantitative analysis revealed that these findings were statistically significant (Figure S3 E and F). These results suggested that the function of *pex16* genes may be evolutionarily conserved between these two species, and an excess amount of PEX16 affects the formation or homeostasis of peroxisomes.

### *pex16* homozygous adult flies showed a reduced body size and rosy eyes

**Figure 2:**
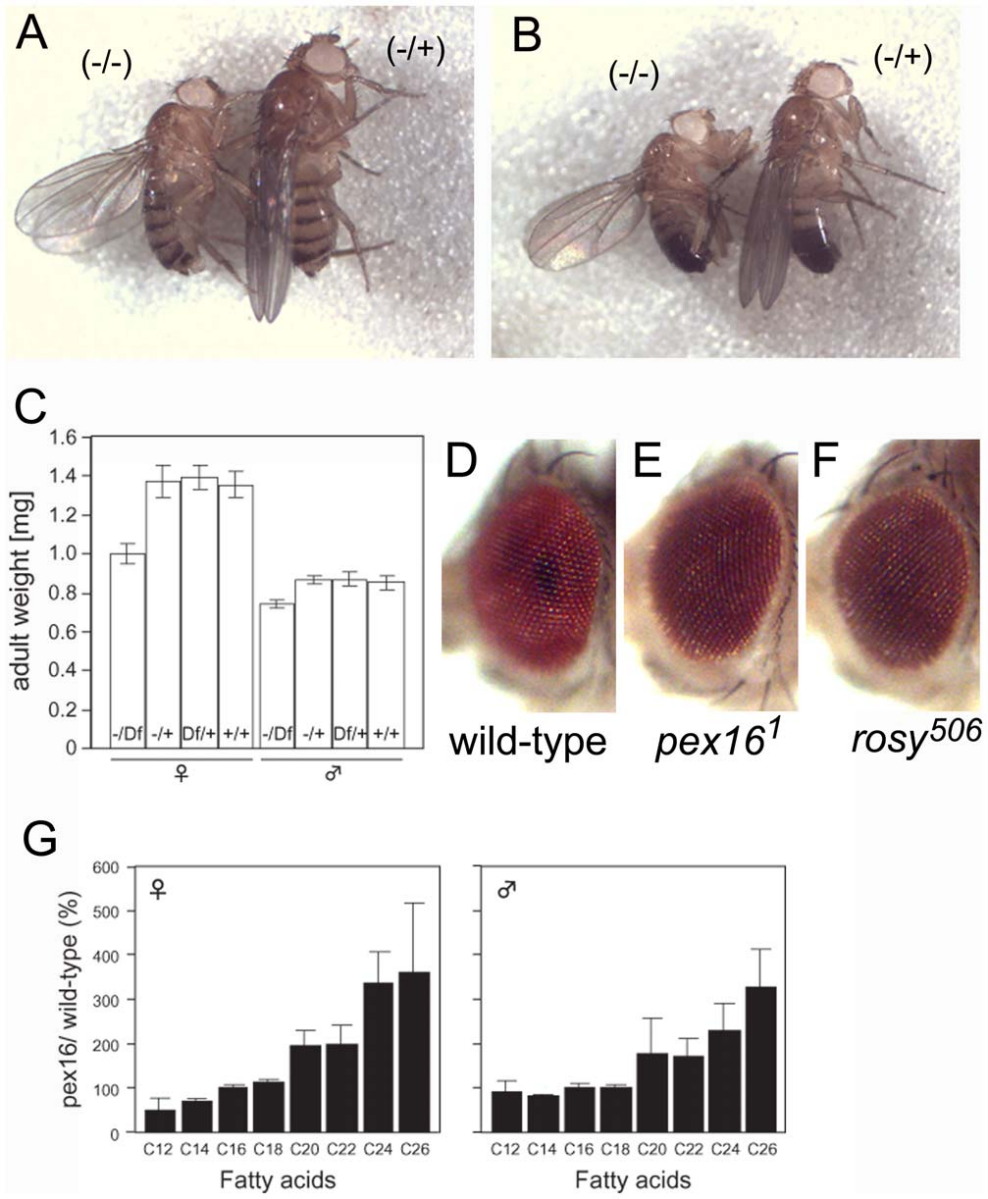
The *pex16* mutant showed a reduced body size, rosy eyes, and the accumulation of VLCFAs. (A and B) The *pex16^1^* homozygote was smaller than wild-type. An adult female (A) and male (B) *pex16^1^* homozygote (−/−) and heterozygote (−/+) are shown. (C) Mean body weight of individual adult flies. Genotype: (−/Df), *pex16^1^*/*Df(3L)ri-XT1*; (−/+), *pex16^1^*/*TM3*; (Df/+), *Df(3L)ri-XT1*/*Dr*; (+/+), *Dr*/*TM3*. Error bars represent standard deviations. (D–F) *pex16* homozygotes had rosy eyes. Eyes of wild-type (D), a *pex16^1^* homozygote (E), and a *rosy^506^* homozygote (F) are shown. (G) VLCFAs accumulated in the *pex16^1^* homozygote. The fatty acid content of adult males homozygous for *pex16* was measured by gas chromatography three days after eclosion. Each bar represents the average level of each VLCFA component in *pex16^1^* homozygotes compared to that in wild-type flies, obtained from three independent experiments. Error bars represent standard deviations.

Severe clinical types of PBDs, including Zellweger syndrome, result in infant death [2]. However, patients with PBDs also develop postnatal disorders; thus, it is important to understand the mechanisms that cause these defects to arise after birth. Therefore, in this study, we focused on the analysis of *pex16* mutants, which survived to the imago stage. Trans-heterozygotes of *pex16^1^* and Df(3L)ri-XT1, uncovering *pex16* locus, developed into adult at normal ratio. However, eclosed *pex16* mutants appeared smaller than wild-type (Figure 2A and 2B). Mean body weight of *pex16* mutants was reduced by 70% in females and 85% in males compared with those of control flies at the same days (two days) after eclosion (Figure 2C). We could not find impaired feeding activity (data not shown), suggesting that reduced body size in *pex16* mutant is probably not due to their starvation. Adult flies homozygous for *pex16^1^* also showed an eye color phenotype similar to that of *rosy* (Figure 2D–F). The *Drosophila rosy* gene encodes xanthine dehydrogenase, which is involved in the production of drosopterin, a component of eye pigments, and the gene product of *rosy* functions in peroxisomes [28], [32]. This result is consistent with our idea that the functions of peroxisomes were largely abolished in the *pex16^1^* homozygote.

### VLCFAs accumulated in the *pex16^1^* homozygotes

The accumulation of VLCFAs in blood plasma is a diagnostic marker of PBDs [1], [2]. In patients with Zellweger syndrome, the plasma level of VLCFAs is 2-10-fold greater than normal [33]. Therefore, we measured the fatty acid content in an extract of whole flies by gas chromatography. Compared with the levels of fatty acids in wild-type flies, which we defined as 100%, the level of VLCFAs with a chain length great than C_24_ was two-fold higher in the *pex16^1^* homozygotes (Figure 2G). However, fatty acids with chain lengths shorter than C_18_ were not affected in these flies (Figure 2G). Thus, the fatty-acid metabolism was altered in the *Drosophila pex16* mutants, much as it is in PBD patients. It was also reported that VLCFAs are increased in a mutant of *pex10*, required for the peroxisomal matrix protein import, in *Drosophila* [24]. Taken together with our finding that the number of peroxisomes is severely reduced in the *pex16^1^* homozygotes, these results suggest that the *pex16^1^* mutant can serve as a potential *Drosophila* model of Zellweger syndrome, although it does not recapitulate the infant death associated with this syndrome.

### *pex16* homozygotes show locomotion defects

**Figure 3:**
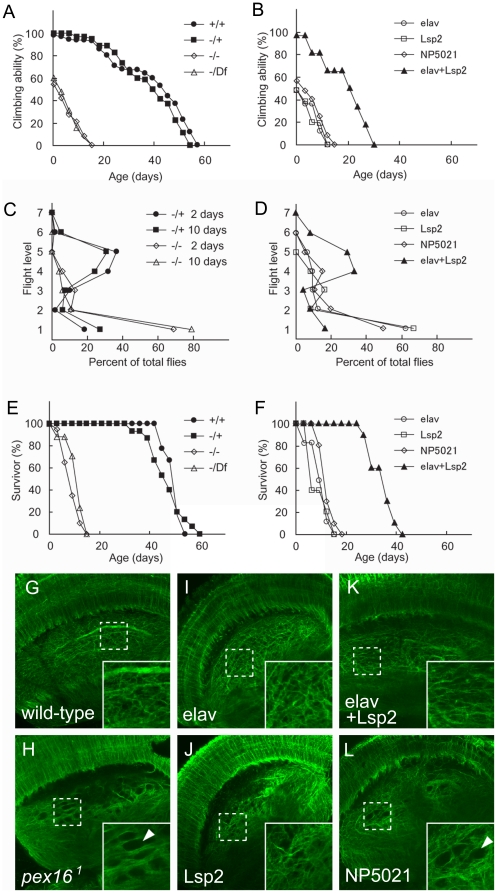
*pex16* homozygotes showed defects in neural functions and development reminiscent of PBD-associated symptoms. (A and B) Climbing assay scores with increasing age (days after eclosion). (A) Genotypes: (+/+), wild-type; (−/+), *pex16^1^*/+; (−/−), *pex16^1^*/*pex16^1^*; (−/Df), *pex16^1^*/ Df(3L)ri-XT1. Averages of three trials are shown. (B) Climbing scores with the wild-type *pex16* gene driven by the indicated GAL4 drivers (key shown at right) in *pex16^1^* homozygotes. GAL4 drivers: elav, elav-GAL4 (expressed in differentiated neurons); Lsp2, Lsp2-GAL4 (expressed in the fat body); NP5021, a GAL4 line expressing GAL4 ubiquitously in the gut. (C and D) Flight activity scores. Fight levels (1–7) plotted against the percentage of flies at each level are shown. Each trial included 20 flies. (C) Genotypes: (−/+), *pex16^1^*/+; (−/−), *pex16^1^*/*pex16^1^*. Flies were examined two (2 days) and ten (10 days) days after eclosion. (D) Flight scores when the wild-type *pex16* gene was driven by the indicated GAL4 drivers in *pex16^1^* homozygotes (see description of drivers in B). Flight activities were measured two days after eclosion. (E and F) Longevity of adult male flies. Percentage of survivors (n  =  20) at 25°C was determined every three days. (E) Genotypes were as described in A. (F) Longevity when the wild-type *pex16* gene was driven by the indicated GAL4 drivers (described in B) in *pex16^1^* homozygotes. (G–L) *pex16^1^* homozygotes showed defective neural development. Brains from adult male flies one day after eclosion were stained with the 22C10 antibody to reveal the axonal and dendritic structures. Confocal images of the optic lobe are shown: wild-type (G), *pex16^1^* homozygote (H), *pex16^1^* homozygote overexpressing wild-type *pex16* driven by elav-GAL4 (I), Lsp2-GAL4 (J), elav-GAL4 and Lsp2-GAL4 (K), or NP5021 (L). Insets are higher magnifications of the areas enclosed by broken lines. Arrowheads indicate spongy structures.

Patients with PBDs exhibit a range of neurological abnormalities [1], [2], including motor dysfunctions. The *Drosophila pex* gene mutants previously reported show defects in spermatogenesis and fatty acid metabolism, but they do not show other phenotypes reminiscent of the symptoms found in PBDs, such as locomotion defects and a shortened lifespan [24]. Hence, we first studied two different locomotion behaviors in adult *pex16^1^* homozygotes: climbing [34], [35] and flight [36], [37]. Wild-type adults have a strong negative geotactic response. Flies tapped to the bottom of a vial quickly climb the wall and tend to stay near the top [34], [35]. We found that *pex16^1^* homozygotes climbed less actively, even immediately after eclosion (Figure 3A). Furthermore, their performance declined more rapidly with age than that of wild-type flies (Figure 3A).

To test the flying ability of *pex16^1^* homozygotes, we used the sticky-cylinder assay [36], [37]: when flies are dropped into a cylinder whose inside is sticky, those with greater flying ability tend to stick higher up on the cylinder wall. In this assay, most of the flies heterozygous for *pex16^1^* stuck to the wall between levels 4 and 5 (Figure 3C). In contrast, *pex16^1^* homozygotes tended to stick at the lowest level (Figure 3C). Both locomotion defects were rescued by the simultaneous tissue-specific expression of *pex16* in the fat body (driven by Lsp2-GAL4) and differentiated neurons (driven by elav-GAL4), but not by its expression in either tissue alone (Figure 3B and 3D). In contrast, the overexpression of *pex16* in other tissues, including the gut, malpighian tubules, and salivary glands, did not rescue these defects (Figure 3B and 3D). Therefore, these locomotion defects are attributable to multiple causes associated with the absence of peroxisomes.

In addition to the defects in neural functions, the lifespan of *pex16^1^* homozygotes was severely reduced. The mean longevity of the females was reduced to one-third and that of the males to one-fourth of the wild-type lifespan (Figure 3E and data not shown). The shortened lifespan of the *pex16^1^* homozygotes was also rescued by the simultaneous overexpression of *pex16* in the fat body and differentiated neurons (Figure 3F). Since the locomotion defects and reduced lifespan of the *pex16^1^* homozygotes were both rescued by the overexpression of *pex16* in the same tissues, we speculate that the short lifespan of the *pex16^1^* homozygotes was caused by their locomotion defects.

### *pex16* homozygotes show defects in nervous system development

Among the neurological abnormalities of PBD, these patients show structural defects of the nervous system [1]. We therefore examined whether the *Drosophila pex16* mutants also showed structural abnormalities in the adult brain. We found a structural abnormality of the dendritic trees in the lobula plate of the optic lobe (Figure 3G and 3H). In the wild-type brain, the dendritic trees were densely distributed (Figure 3G). In contrast, in the *pex16^1^* homozygotes one day after eclosion, areas of low-density dendrites were observed (Figure 3H), whereas other parts of the brain appeared normal (data nor shown). The glial cells and presynaptic structures in the optic lobe also appeared to be unaffected (Figure S4). The reduction of dendrites in the *pex16^1^* homozygotes was already detectable at the pupal stage, and the defect had not worsened at 10 days after eclosion (data not shown), suggesting that this abnormality was associated with developmental defects instead of neuronal degeneration. In addition, we did not find that this defect worsened with age (data not shown).

We also found that the abnormality of the lobula plate was rescued by the tissue-specific expression of *pex16* either in the fat body (driven by Lsp2-GAL4) or differentiated neurons (driven by elav-GAL4) (Figure 3I and 3J). These results indicate that the developmental defects in neurons that were caused by the absence of peroxisomes could be rescued by the functions of peroxisomes in different organs. Considering that the locomotion defects of the *pex16^1^* homozygote were rescued by the simultaneous tissue-specific expression of *pex16* in the fat body and differentiated neurons, but not by its expression in either tissue alone (Figure 3B and 3D), we speculated that the observed defect in the lobula plate is not a cause of the locomotion abnormalities in the *pex16^1^* homozygote. This idea is also consistent with our finding that the locomotion defects progressed with age in adult flies, whereas the structural defect of the lobula plate did not.

### Maturation of male germ cells is arrested in the testes of *pex16* homozygotes

**Figure 4:**
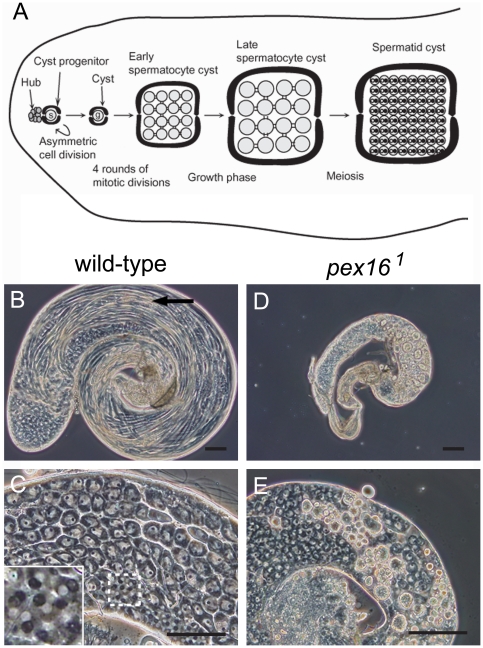
The maturation of germline cells is arrested in the testis of *pex16* homozygotes. (A) Schematic representation of early spermatogenesis. Germline stem cells (s) and somatic cyst progenitor cells, anchored to somatic hub cells, produce a new stem cell and a gonialblast (g), which are enclosed by two somatic cyst cells (Cyst). A single gonialblast cell undergoes four rounds of mitotic divisions to produce 16 early spermatocytes that then enter the growth phase. After the growth phase, the late spermatocytes undergo two meiotic divisions, producing 64 haploid spermatids. (B–E) Phase-contrast micrographs of the wild-type (B and C) and *pex16^1^* homozygote (D and E) testes. The bundles of elongated spermatids (arrow in B) and postmeiotic spermatids (inset in C) were missing in the *pex16^1^* homozygote testis. Inset in C is a higher magnification of the area enclosed by broken lines. Scale bars represent 100 µm.

**Figure 5:**
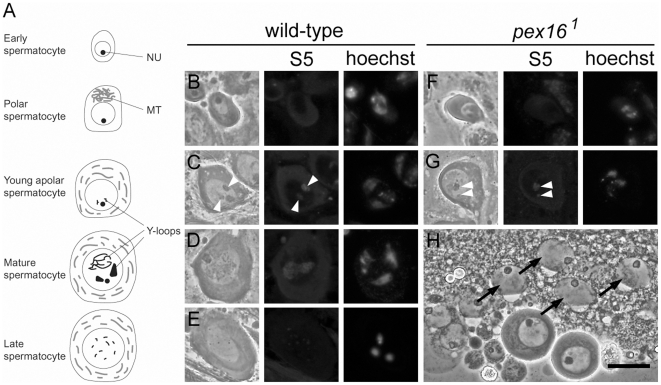
Maturation of spermatocytes is arrested in the spermatocyte growth phase. (A) Schematic representation of spermatocyte growth phase. Nu, nucleolus; MT, mitochondria. (B–G) Maturation of spermatocytes in the testes of wild-type (B–E) and *pex16^1^* homozygote (F–G) flies. Y-chromosome loops and chromatin were stained with the S5 antibody (center panels) and Hoechst 33258 (right panels), respectively. Polar spermatocytes (B and F) and young apolar spermatocytes (C and G) were observed in both wild-type and *pex16^1^* mutant testes, whereas mature spermatocytes (D) and spermatocytes with meiotic chromosome condensation (E) were observed only in wild-type testes. Degenerated spermatocytes (arrows in H) were observed in the *pex16^1^* homozygote testes in squash preparations of live cells. Scale bar represents 20 µm.

In addition to the above-described phenotypes, which showed obvious similarities to the well-characterized symptoms of patients with PBDs, *pex16^1^* homozygotes exhibited male sterility. Because patients with Zellweger syndrome die well before reaching sexual maturity, this possible role of peroxisomes has not been studied in humans. In the *Drosophila* testis, cells in each stage of spermatogenesis can be observed at once (Figure 4A). In wild-type testes, the gonialblast is formed by the asymmetric division of a germline stem cell. Each gonialblast undergoes four rounds of mitotic division to produce 16 early spermatocytes. After this period of mitotic proliferation, each spermatocyte enters a growth phase, which is accompanied by sequential morphological changes as the cells differentiate from the early spermatocyte to the late spermatocyte stage (Figure 5A).

The *pex16^1^* homozygote testis was smaller than the wild-type testis (Figure 4B) and did not contain mature sperm cells (Figure 4D). However, early spermatocyte cysts, which are composed of 16 spermatocytes and two cyst cells, were found in these testes (data not shown), which suggests that the mitotic division of the gonialblast occurred normally in the *pex16* mutant testes. However, we found neither normal postmeiotic spermatids nor elongated spermatids in the mutant testes (Figure 4D and 4E), although these cells were abundant in the wild-type testes (Figure 4B and 4C). These results suggested that the maturation of spermatocytes is arrested during the growth phase in the *pex16* mutants.

Next, we examined the maturation of the *pex16* mutant spermatocytes in detail (Figure 5A) [38]. Early spermatocytes develop into polar spermatocytes, whose mitochondria form a cluster in the cytoplasm that is distributed asymmetrically within the cell (Figure 5B). These cells then develop into young apolar cells, in which the mitochondria disperse uniformly into the cytoplasm (Figure 5C). In the young apolar stage, phase-dark bodies that correspond to the primordia of *kl-5* and *ks-1* Y- chromosome loops appear in the nucleus (Figure 5C) [39], [40]. These chromosome loops can be detected by immunostaining with the S5 antibody, which is directed against a nascent RNA-associated protein (Figure 5C) [41]. In the mature spermatocytes, the *kl-3* loop is also visible, and the Y chromosome loops occupy most of the nucleus (Figure 5D). The end of the growth phase is marked by the disintegration of the Y loops and nucleolus (late spermatocyte phase), and chromosome condensation is visible at the beginning of meiosis (Figure 5E).

To determine when the development of the spermatocytes is arrested in the testes of the *pex16^1^* homozygotes, we analyzed their growth phases. We identified polar spermatocytes based on their morphology (Figure 5F) and young apolar spermatocytes in which the primordia of *kl-5* and *ks-1* Y chromosome loops were visible (Figure 5G). However, we did not find any mature spermatocytes or late spermatocytes, although they were present in the wild-type testes (Figure 5D and 5E). Instead, degenerated spermatocytes, which could be identified by the absence of most of the cytoplasm, were present in the *pex16* homozygote testes (Figure 5H). Therefore, the maturation of the spermatocytes was arrested at the young apolar stage in the testes of *pex16* homozygotes. These results were consistent with those previously observed in other *Drosophila pex* mutants [24].

### Peroxisomes in somatic cyst cells are required for germ-cell maturation

**Figure 6:**
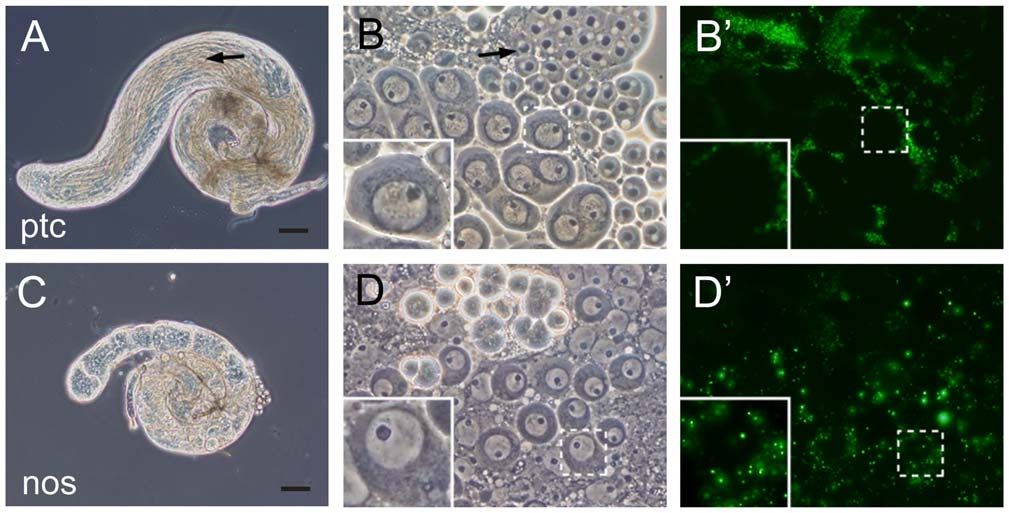
Peroxisomes in somatic cyst cells are essential for male germ cell maturation. (A–D) Phase-contrast micrographs of a *pex16^1^* homozygote testis expressing *UAS-pex16* and *UAS-EGFP-SKL* driven by *ptc-GAL4*, expressed in cyst cells (A and B) or *nos-GAL4*, expressed in germline cells (C and D). Elongated spermatids (arrow in A) and morphologically normal spermatids (arrow in B) are indicated. (B′ and D′) Peroxisomes detected by EGFP-SKL. The fluorescent images in B′ and D′ correspond to the phase-contrast micrographs of B and D, respectively. Insets showing single spermatocytes are higher magnifications of the areas enclosed by broken lines. Scale bars represent 100 µm.

The spermatocyte cyst of *Drosophila* is composed of spermatocyte and cyst cells (Figure 4A), and peroxisomes were present in both cell types in the wild-type testes (Figure S5A and S5E). On the other hand, in the testis of *pex16^1^* homozygotes, peroxisomes were absent from the spermatocytes and cyst cells, although the cyst cells were morphologically normal (Figure S5B, S5D, and S5F). Therefore, we determined whether peroxisomes were required in the spermatocytes or the cyst cells for spermatocyte maturation. We overexpressed the *pex16* gene specifically in cyst cells (driven by ptc-GAL4) or germline cells (driven by nos-GAL4) in the *pex16^1^* mutant background. Peroxisomes were detectable in the cells overexpressing *pex16* (Figure 6B′, D′ and Figure S5). When *pex16* was overexpressed in cyst cells, but not germline cells, the fertility of the *pex16^1^* males was rescued (data not shown). Morphologically normal spermatids and moving mature sperm were found in these flies, even thought the germ cells lacked peroxisomes (Figure 6A and 6B). In contrast, *pex16* expression in germ cells did not rescue the sterility or defective spermatogenesis of the *pex16^1^* homozygotes (Figure 6C and 6D). These results indicated that peroxisomal functions in somatic cyst cells were essential for spermatogenesis, whereas those in germ cells were dispensable. Interestingly, this finding was not comparable with the previously reported results that *Drosophila pex2* in germ cells, but not in cyst cells, is required for germ-cell maturation [24]. This discrepancy may suggest that *pex2* and *pex16* have distinct roles in germ-cell maturation.

## Discussion

In this study, we developed *Drosophila* models of PBDs, in which *Drosophila pex3* or *pex16* genes were disrupted. Based on the molecular lesions of these mutant loci, the *pex3* and *pex16* mutations were null alleles. We found that *pex3^1^* homozygosis caused larval lethality, and peroxisomes were not detected in the cells of the larvae. On the other hand, *pex16^1^* homozygotes were viable, and a very small number of peroxisome-like particles were still observed in their cells. These differences could be attributed to distinct requirements for PEX3 and PEX16 in the formation of the peroxisome membrane in *Drosophila*.

Studies using human cells from PBD patients show that PEX3 and PEX16 are indispensable for the PMP sorting pathway, which is essential for the formation of the peroxisome membrane [20], [21]. However, the necessity of PEX16's contribution to PMP sorting seems to vary among species, although the role of PEX3 is probably conserved evolutionarily. Most yeast species do not have a *Pex16* orthologue, with the exception of *Yarrowia lipolytica*, whose *Pex16* orthologue is *Pex16p* [42]. In *Yarrowia lipolytica*, Pex16p is involved in the import of peroxisome matrix proteins and peroxisome proliferation, but not in peroxisome membrane biogenesis [29]. Here we showed that *Drosophila* PEX16 is closely related to yeast Pex16p. Considering that PMP sorting is thought to be essential for the synthesis of peroxisomes, and a small number of peroxisomes are maintained in the absence of PEX16 in yeast and *Drosophila*, some other protein may substitute for PEX16 in the PMP sorting pathway in these species. However, the mechanisms of this substitution remain to be understood.

Peroxisomes participate in many aspects of lipid metabolism [9], [10]. Therefore, it is difficult to identify physiological links between peroxisomal dysfunctions and the pathology of PBDs. In adrenoleukodystrophy (ALD) patients, VLCFA levels in the white matter of the brain correlate with phenotypic severity, so the abnormal accumulation of VLCFAs is thought to be the cause of the neuronal damage associated with ALD [43]. In *Drosophila*, homozygotes for *pex2* or *pex10* also show the accumulation of VLCFAs [24], and the high level of VLCFAs was suggested to be responsible for the defective spermatogenesis in these mutants [24]. However, these flies do not show other phenotypes reminiscent of PBDs, including neuronal function disorders [24]. These results suggest that the accumulation of VLCFAs may not be the cause of the neuronal function disorders in *Drosophila*. On the other hand, we found that *pex16* homozygotes demonstrate phenotypes that have some homology to the disorders found in Zellweger syndrome patients, including a reduced lifespan, locomotion defects, and abnormal neuronal development, with some exceptions such as infant death. Given that none of these phenotypes is found in the homozygotes for *pex2* or *pex10* [24], abnormalities in other lipids metabolized in peroxisomes besides VLCFAs could contribute to these disorders. Animal models of PBDs, including our *Drosophila* models, may aid in identifying the causes of these diseases.

Our results showed that peroxisomes were essential for the maturation of male germline cells in *Drosophila*, although their presence was required in the somatic cyst cells, rather than in the spermatocytes. These results are not consistent with the previous report that *pex2* in germline cells but not in cyst cells is required for spermatogenesis in *Drosophila* [24]. Three RING proteins, Pex2, Pex10, and Pex12 in yeast are involved in the ubiquitination of PTS1 receptor Pex5 and contribute to PTS1-dependent peroxisomal matrix protein import [44]. However, the *pex10* in germ cells is not required for spermatogenesis, although mutations of *pex2* and *pex10* result in similar spermatogenesis defects in *Drosophila* [24]. Based on these results, it was suggested that *pex2* and *pex10* have different roles in spermatogenesis [24]. Therefore, it is possible that the roles of PEX2 in spermatogenesis are not associated with peroxisomes, although there may be other explanations.

Our results demonstrate that a function of peroxisomes in the somatic cyst cells is essential for the maturation of adjacent germline cells. This cell non-autonomous function of peroxisomes suggests that some signal directly or indirectly dependent on peroxisome functions is sent from the cyst cells to the spermatocytes, although we cannot exclude the possibility that peroxisomes suppress the production of toxic substances in cyst cells that inhibit the progression of the spermatocyte growth phase, when the peroxisomes are absent. During the spermatocyte growth phase, a subset of genes are actively transcribed in spermatocytes, and their mRNAs are stored in the cells until after meiosis [45], [46]. Therefore, it is conceivable that a signal from the cyst cells could cell non-autonomously regulate this process. However, the role of cyst cells in the spermatocyte growth phase has not been studied well, and no signal from cyst cells that affects spermatocytes has been described. Our study suggests, however, that such a signal may exist.

## Materials and Methods

### Fly stocks

Fly stocks were maintained at 25°C on a standard cornmeal media. Canton-S and *white^1118^* were used as wild-type controls. The GS9829 and GS14106 lines were used, respectively, to generate the *pex3* and *pex16* mutants [47]. Df(3L)ED218 and Df(3L)ri-XT1 are deletions uncovering the *pex3* and *pex16* loci, respectively. The following *GAL4* driver strains were used: nos-GAL4 expresses GAL4 in germline cells [48]; ptc-GAL4 [49] express GAL4 in cyst cells; Lsp2-GAL4 expresses it in the fat body [50]; elav-GAL4 expresses it in all differentiated neurons; and NP5021 drives GAL4 expression ubiquitously in the gut [51].

### Construction of UAS-*pex16*

To generate UAS-*pex16*, the sole intron in the *pex16* locus was removed by a PCR-based method, and the resulting fragment, containing the entire coding region of *pex16*, was cloned into the *Not*I and *Xho*I sites of pUAST [27]. Transgenic fly lines expressing *UAS-pex16* were generated using a standard procedure.

### Construction of UAS-EGFP-SKL and UAS-PMP70-ECFP

To generate UAS-EGFP-SKL, EGFP was amplified by PCR using the following primers: forward, CCGAATTCACCATGGTGAGCAAGGGCGAGG; reverse, GCCTCGAGTTACAGCTTGCTCTTGTAGCTGCGCTTGTACAGCTCGTCCATGC. The reverse primer encodes a PTS1 (C-terminal SKL sequence). The resulting fragments were digested by *Eco*RI and *Xho*I, and cloned into the *Eco*RI and *Xho*I sites of pUAST. To generate UAS-PMP70-ECFP, a cDNA fragment of PMP70 (CG12703), whose stop codon was removed, was amplified by PCR using LD11581 as a template and cloned into the *Eco*RI and *Bam*HI sites of pECFP-N1 (Clontech). The PMP70-ECFP fragment was re-cloned into the *Eco*RI and *Not*I sites of pUAST [27]. Transgenic lines of UAS-EGFP-SKL and UAS-PMP70-ECFP were generated using standard procedures.

### RT-PCR

Total mRNA was extracted from whole flies using the SV Total RNA Isolation System (Promega). cDNA was synthesized with random hexamer primers using the PrimeScript 1st strand cDNA Synthesis Kit (Takara). The following oligonucleotides were used as PCR primers: F1, 5′-CACGTTATGCACAACGGCG-3′; R1, 5′-CAGCTCGTCCGTGCTGC-3′; F2, 5′-CGCAGCAAGAACAGAGCG-3′; R2, 5′-GCGAAGTGGTATCGAAGCC-3′; F3, 5′-CCACAGCCAAGTGGGTATCC-3′; R3, 5′-CTCCTCGGAATTGCGTGCC-3′; F4, 5′- GCTAAGGTGGTTGCCAAACCC-3′; R4, 5′-CCTCAAGAGCGGCAATGC-3′. The RT-PCR product of Ribosomal protein L32 (RpL32), as a positive control, was obtained using the primers, 5′-CCAAGCACTTCATCCGCCACC-3′ and 5′-GCGGGTGCGCTTGTTCGATCC-3′. The RT-PCR products were analyzed by 1% agarose gel electrophoresis.

### Measurement of adult fly weight

The mean body weights of male and female flies (10–20) were measured two days after eclosion. Flies were reared under the same growth conditions, and flies obtained from three independent cultures were used for the measurement.

### Measurements of locomotor activities

The climbing assay [34], [35]. Flight activity measurement [36] were performed essentially as described previously. Briefly, a measuring cylinder (7-cm diameter and 32-cm height) with a funnel affixed to the top by inserting the stem through a hole drilled in a tight-fitting rubber stopper was used as a flight tester. The inside wall of the tester was coated with liquid paraffin. Ten-twenty flies of the same age were dropped through the funnel and scored immediately according to where they stuck on the cylinder wall, as follows. The wall of the tester was marked every 6 cm from the bottom, to indicate scoring levels. The bottom of the cylinder was defined as 1. Flies that landed above level 6 were given the score of 7.

### Histological analysis of the brain

The brain of adult or pupal flies was dissected in PBS (137 mM NaCl, 2.68 mM KCl, 10.14 mM Na_2_HPO_4_, 1.76 mM KH_2_PO_4_), fixed with 4% paraformaldehyde in PBS at 4°C for 2 hr, and stained with an antibody to microtubule-associated protein 1B (22C10, 1∶5 dilution, DSHB), anti-Bruchpilot (nc82, 1∶20 dilution, DSHB), anti-Repo (8D12, 1∶20 dilution, DSHB), or anti-Homer [52] (1∶200 dilution), and an Alexa Fluor 488 Goat Anti-mouse IgG secondary antibody (1∶100 dilution, Invitrogen). The samples were observed using an LSM5 confocal microscope (Zeiss).

### Detection of peroxisomes in S2 cells and *in vivo*

*Drosophila* S2 cells were cultured and transfected as described previously [53]. The slide was treated with 0.5 mg/ml concanavalin A (Sigma) and allowed to air-dry before the cells were transferred to it. The cells were observed using an LSM5 confocal microscope (Zeiss).

To visualize EGFP-SKL in the malpighian tubules and testes, these tissues were dissected in PBS, fixed in 4% paraformaldehyde, and stained with an anti-GFP antibody (1∶100 dilution, Nakalai Tesque) and an Alexa Fluor 488 Goat Anti-rat IgG secondary antibody (1∶100 dilution, Invitrogen).

### Quantitative analysis of peroxisome number

In each confocal image of Malpighian tubule cells and spermatocytes, the number of peroxisomes, detected by EGFP-SKL, per 1,000 µm^2^ cytoplasmic region was counted. In addition, the size of the peroxisomes was estimated from the number of pixels comprising each peroxisome in confocal images using the Photoshop histogram function. A peroxisome with over twice the pixel number compared with the average pixel number of wild-type peroxisomes was defined as an enlarged peroxisome.

### Histological analysis of the testes

To observe the maturation of spermatocytes, the testes of newly eclosed male flies were examined by phase-contrast light microscopy (Zeiss). To observe the Y-chromosome loops in the spermatocytes, the testes were squashed on a slide glass, and the spermatocytes were frozen in liquid nitrogen and fixed with methanol-acetone as described [54]. The fixed spermatocytes were stained with an anti-S5 antibody (1∶20 dilution) [41] and a Cy3-conjugated Donkey Anti-mouse IgG secondary antibody (1∶100 dilution, Jackson IR).

### Electron microscopy

The malpighian tubules were fixed with 2% glutaraldehyde in 0.1 M sodium cacodylate (pH 7.4) for 1 hr on ice. Then peroxidase cytochemistry was performed as described [28] [55]. After the peroxidase reaction, the specimens were postfixed with 1% OsO_4_ for 1 hr, dehydrated, and embedded in Epon as described elsewhere [56].

### Measurement of fatty acids

Lipids of whole male or female flies were extracted according to a method described previously [57]. The extracted lipids were treated with a dehydrated methanol∶acetyl chloride mixture (10∶1) to extract the fatty acid methyl esters. The fatty acid methyl ester derivatives were analyzed on a GC 353B gas chromatograph equipped with a flame ionization detector (GL Sciences, Tokyo, Japan) and a TC-FFAP capillary column (60 m×0.25 mm internal diameter, 0.25 µm; GL Sciences). The oven temperature was programmed to increase from 170°C to 230°C at 5°C/min followed by a hold of 15 min. The injector and detector temperatures were both set at 250°C. Helium was used as the carrier gas, at 1 ml/min. Individual fatty acids were identified by comparing the retention times with those of known fatty acid standards.

## Supporting Information

Figure S1 **Full-length mRNAs are not synthesized from** ***pec3^1^*** **and** ***pex16^1^*** **mutant loci.** RT-PCR was performed using wild-type (wt), *pex3^1^* homozygote, and *pex16^1^* homozygote template DNA. RT-PCR products were obtained using the indicated primers (F1, R1, F2, R2, F3, R3, F4, and R4, whose location in the *pex3^1^* and *pex16^1^* loci are shown by arrows. RpL32 was used as a positive control. The sizes of the PCR products were 152 base pairs (bp) (RpL32), 179 bp (F1+R1), 180 bp (F2+R2), 200 bp (F3+R3), and 284 bp (F4+R4). Scale bar represents 0.5 Kb.
(TIF)

Figure S2 **EGFP-SKL is co-localized with PMP70-ECFP in S2 cells.** Peroxisomes were detected in S2 cells by EGFP-SKL (green in left panel) and PMP70-ECFP (magenta in middle panel). The merged image is shown at right.
(TIF)

Figure S3 **Overexpression of** ***pex16*** **results in fewer but enlarged peroxisomes.** UAS-EGFP-SKL was driven by NP5021 (for expression in whole gut, A) or nos-GAL4 (for expression in germ cells, C). Both UAS-pex16 and UAS-EGFP-SKL were driven by NP5021 (B) or nos-GAL4 (D). Malpighian tubules (A and B) and spermatocytes (C and D) are shown. The fluorescent images in A′ to D′ correspond to the phase-contrast micrographs in A to D, respectively. Arrowhead in D′ indicates an enlarged peroxisome. Scale bars represent 20 µm (A′) and 10 µm (C′). (E and F) Average number of peroxisomes per 1,000 µm^2^ of cytoplasm in the confocal images of malpighian tubule cells (E) and spermatocytes (F). Magenta in F indicates the number of enlarged peroxisomes, which were defined as being over twice the average size of wild-type peroxisomes.
(TIF)

Figure S4 **Presynaptic structures and glial cells appear unaffected in** ***pex16^1^*** **flies.** (A and B) Brains from adult flies were stained with an nc82 antibody to observe presynapses. Confocal images of the optic lobe are shown: wild-type (A) and *pex16^1^* (B). (C and D) Brains from adult flies were stained with anti-Repo (Green) and anti-Homer (Magenta) antibodies to show glial cells and neuropile, respectively. Projection images of the optic lobe are shown: wild-type (C) and *pex16^1^* (B). Scale bars represent 20 µm.
(TIF)

Figure S5 **Cyst cells form normally in the testis of** ***Pex16^1^*** **homozygotes.** (A and B) Peroxisomes were detected in the spermatocytes or cyst cells of the wild-type testes (A), but not in the *pex16^1^* homozygote (B) testes expressing *UAS-EGFP-SKL* driven by *Act-GAL4*. Scale bar in A represents 100 µm. (C and D) The cyst cells were morphologically normal in the testes of *pex16^1^* homozygotes. *UAS-GFP* was driven by *ptc-GAL4*, expressing GAL4 in cyst cells, in the testes of wild-type (C) and *pex16^1^* homozygotes (D). Cyst cells were detected by anti-GFP antibody staining and are indicated by white arrowheads in C and D. (E and F) Peroxisomes were absent in the spermatocytes of *pex16^1^* homozygotes. Phase-contrast micrographs of spermatocytes in wild-type (E) and *pex16^1^* homozygote (F) testes expressing *UAS-EGFP-SKL* driven by *nos-GAL4* are shown. The fluorescent images shown in E′ and F′ correspond to the phase-contrast micrographs of E and F, respectively. Scale bar in E represents 20 µm.
(TIF)
