# Supplementary figures and images for: Drosophila Carrying Pex3 or Pex16 Mutations Are Models of Zellweger Syndrome That Reflect Its Symptoms Associated with the Absence of Peroxisomes
Source: PLoS One. 2011 Aug 3;6(8):e22984. doi: 10.1371/journal.pone.0022984 (PMC3149631; doi:10.1371/journal.pone.0022984)

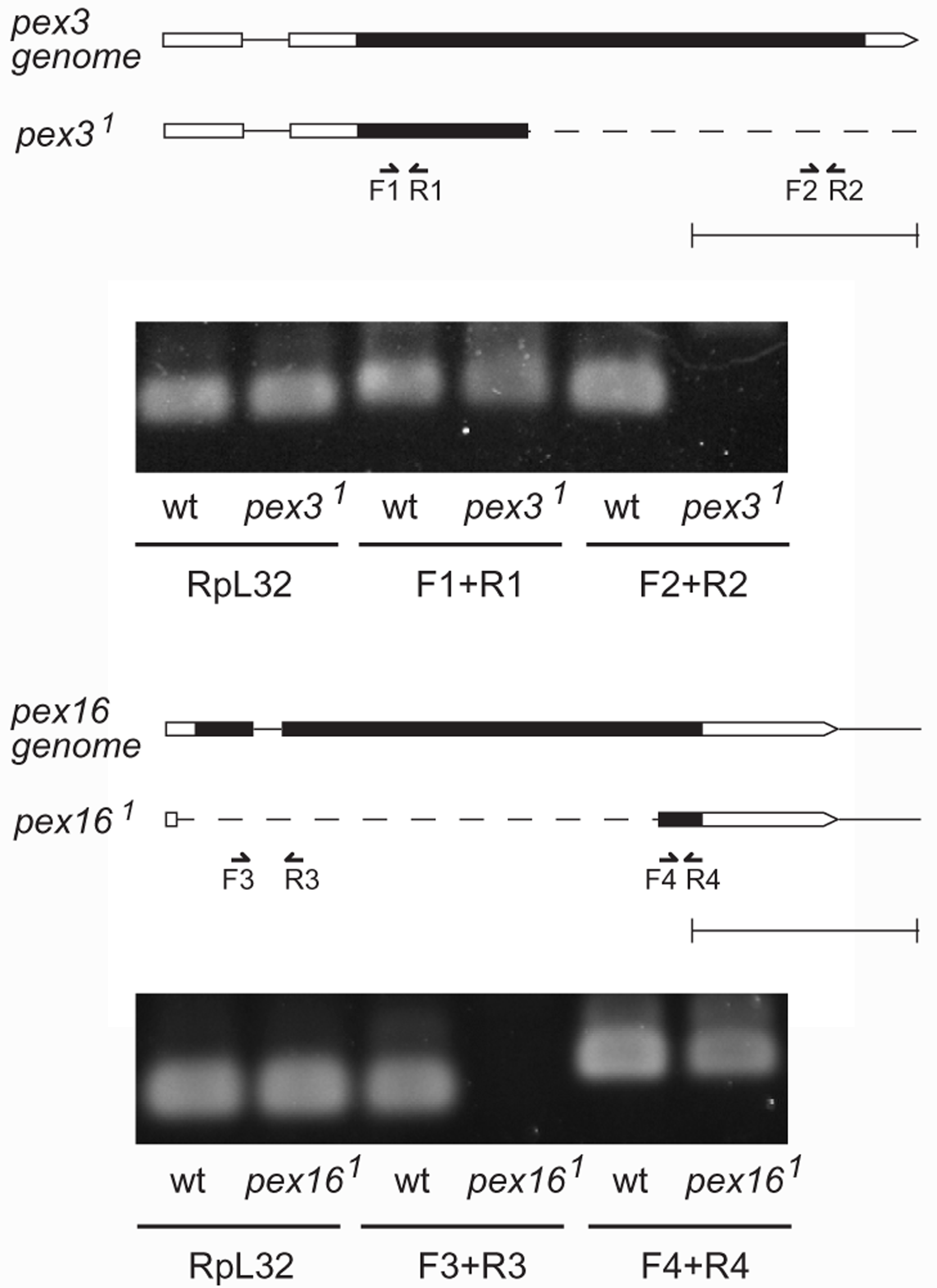

Supplement: Figure S1 — Full-length mRNAs are not synthesized from pec31 and pex161 mutant loci. RT-PCR was performed using wild-type (wt), pex31 homozygote, and pex161 homozygote template DNA. RT-PCR products were obtained using the indicated primers (F1, R1, F2, R2, F3, R3, F4, and R4, whose location in the pex31 and pex161 loci are shown by arrows. RpL32 was used as a positive control. The sizes of the PCR products were 152 base pairs (bp) (RpL32), 179 bp (F1+R1), 180 bp (F2+R2), 200 bp (F3+R3), and 284 bp (F4+R4). Scale bar represents 0.5 Kb. (TIF) [file pone.0022984.s001.tif]

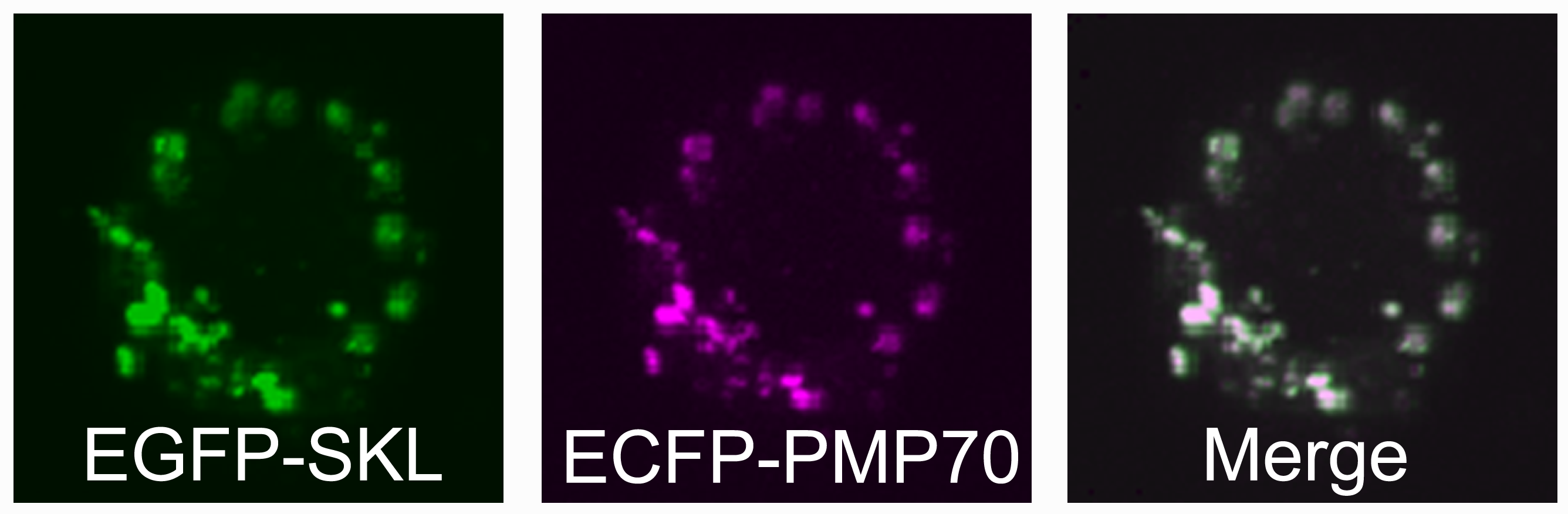

Supplement: Figure S2 — EGFP-SKL is co-localized with PMP70-ECFP in S2 cells. Peroxisomes were detected in S2 cells by EGFP-SKL (green in left panel) and PMP70-ECFP (magenta in middle panel). The merged image is shown at right. (TIF) [file pone.0022984.s002.tif]

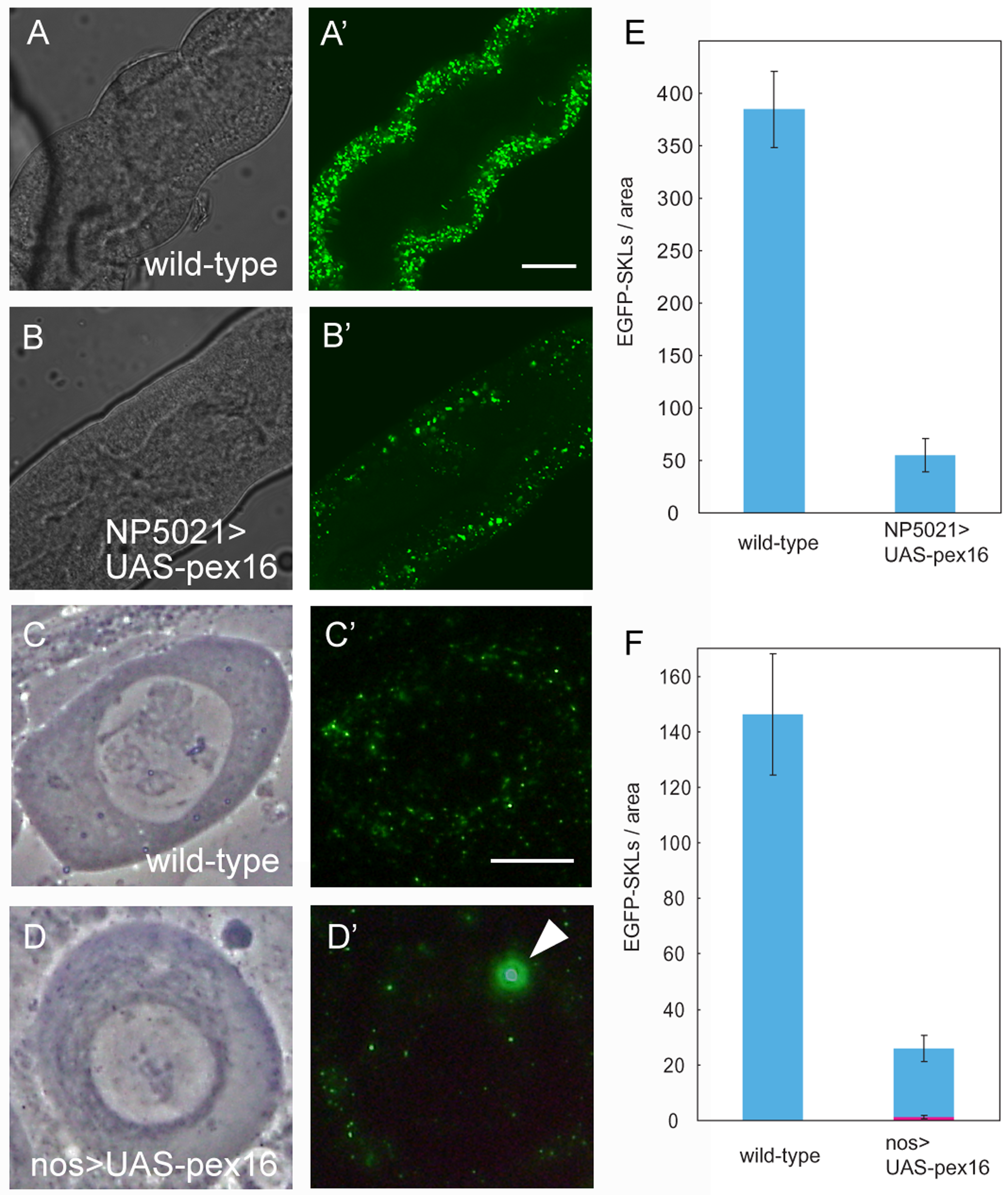

Supplement: Figure S3 — Overexpression of pex16 results in fewer but enlarged peroxisomes. UAS-EGFP-SKL was driven by NP5021 (for expression in whole gut, A) or nos-GAL4 (for expression in germ cells, C). Both UAS-pex16 and UAS-EGFP-SKL were driven by NP5021 (B) or nos-GAL4 (D). Malpighian tubules (A and B) and spermatocytes (C and D) are shown. The fluorescent images in A′ to D′ correspond to the phase-contrast micrographs in A to D, respectively. Arrowhead in D′ indicates an enlarged peroxisome. Scale bars represent 20 µm (A′) and 10 µm (C′). (E and F) Average number of peroxisomes per 1,000 µm2 of cytoplasm in the confocal images of malpighian tubule cells (E) and spermatocytes (F). Magenta in F indicates the number of enlarged peroxisomes, which were defined as being over twice the average size of wild-type peroxisomes. (TIF) [file pone.0022984.s003.tif]

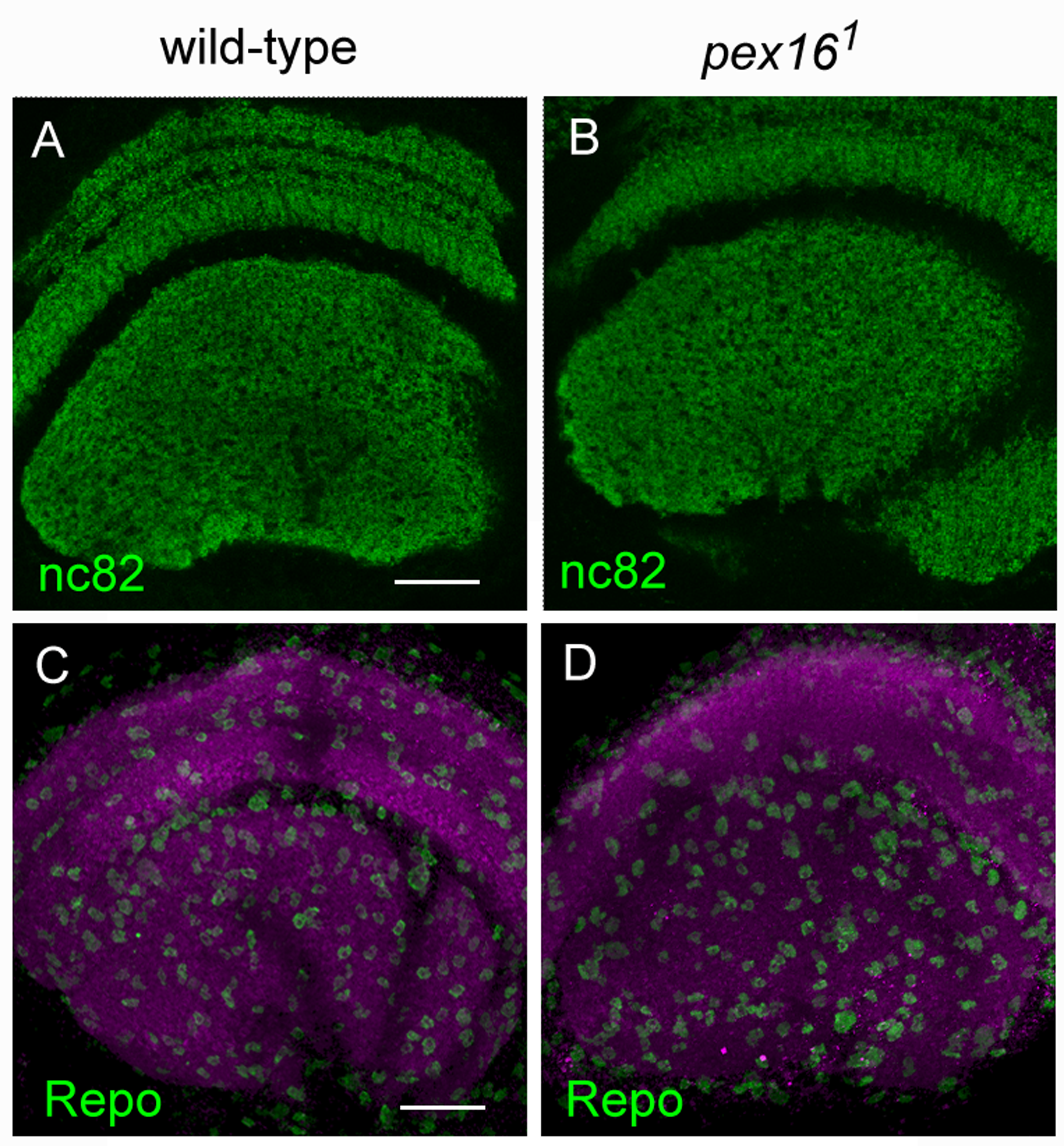

Supplement: Figure S4 — Presynaptic structures and glial cells appear unaffected in pex161 flies. (A and B) Brains from adult flies were stained with an nc82 antibody to observe presynapses. Confocal images of the optic lobe are shown: wild-type (A) and pex161 (B). (C and D) Brains from adult flies were stained with anti-Repo (Green) and anti-Homer (Magenta) antibodies to show glial cells and neuropile, respectively. Projection images of the optic lobe are shown: wild-type (C) and pex161 (B). Scale bars represent 20 µm. (TIF) [file pone.0022984.s004.tif]

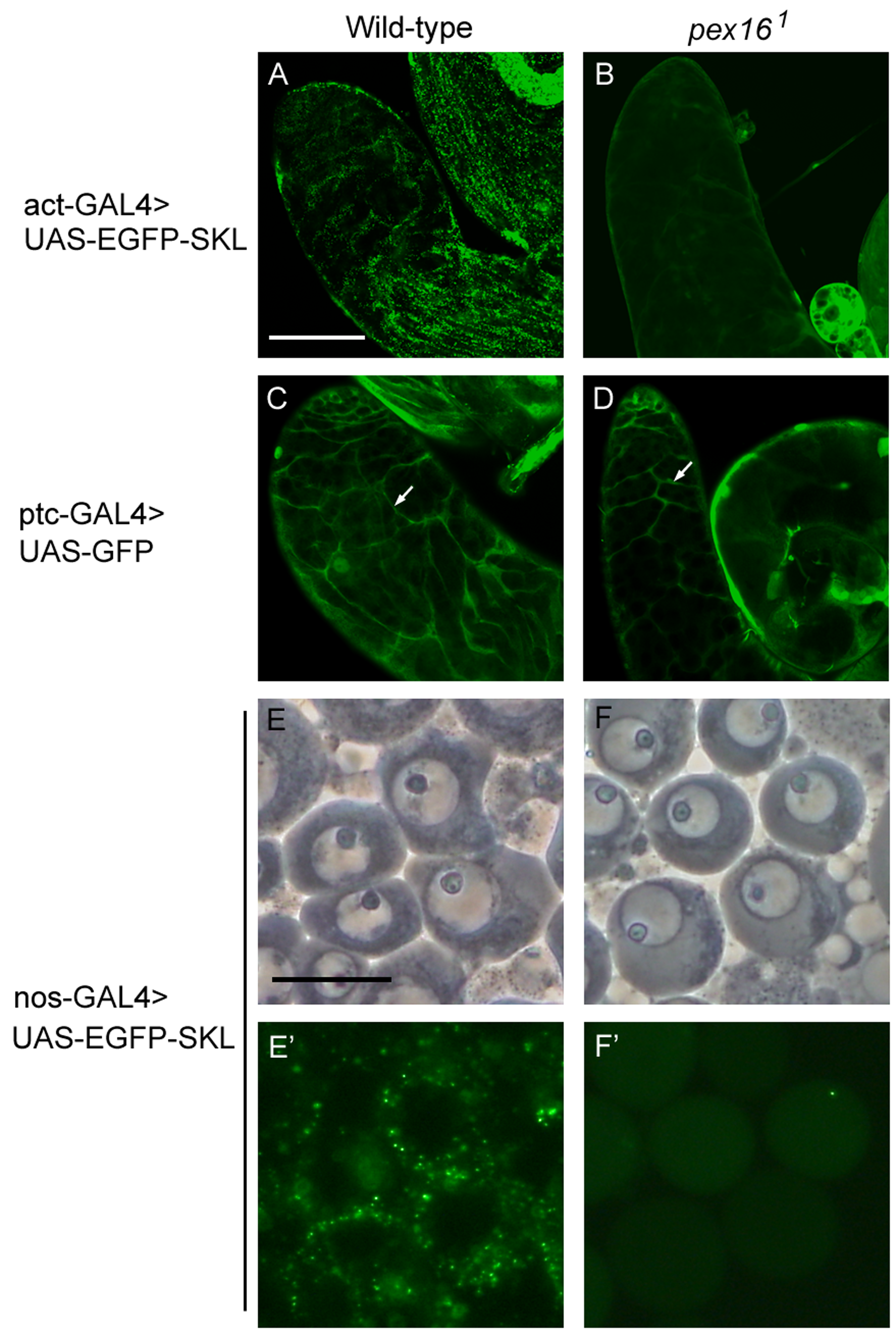

Supplement: Figure S5 — Cyst cells form normally in the testis of Pex161 homozygotes. (A and B) Peroxisomes were detected in the spermatocytes or cyst cells of the wild-type testes (A), but not in the pex161 homozygote (B) testes expressing UAS-EGFP-SKL driven by Act-GAL4. Scale bar in A represents 100 µm. (C and D) The cyst cells were morphologically normal in the testes of pex161 homozygotes. UAS-GFP was driven by ptc-GAL4, expressing GAL4 in cyst cells, in the testes of wild-type (C) and pex161 homozygotes (D). Cyst cells were detected by anti-GFP antibody staining and are indicated by white arrowheads in C and D. (E and F) Peroxisomes were absent in the spermatocytes of pex161 homozygotes. Phase-contrast micrographs of spermatocytes in wild-type (E) and pex161 homozygote (F) testes expressing UAS-EGFP-SKL driven by nos-GAL4 are shown. The fluorescent images shown in E′ and F′ correspond to the phase-contrast micrographs of E and F, respectively. Scale bar in E represents 20 µm. (TIF) [file pone.0022984.s005.tif]
